# Supplementary material for: Patterns of tropical forest understory temperatures
Source: Nat Commun. 2024 Jan 23;15:549. doi: 10.1038/s41467-024-44734-0 (PMC10805846; doi:10.1038/s41467-024-44734-0)
Supplement: Supplementary file 1 — Supplementary Information [file 41467_2024_44734_MOESM1_ESM.pdf]

Supplementary Text

Modelling software and implementation schemes

For this study primarily two software were used MATLAB and R. The ML modelling was done in MATLAB whereas Semivariogram analysis was done in R using 'gstat' package[1]. A 5-fold cross-validation (CV) scheme was used in ML modelling to minimize overfitting or underfitting of the model. Under the 5-fold CV scheme randomly shuffled data is stratified into five groups. For each group, data is further divided into 5 subsets. One subset is used for testing and remaining is used for training of the model. This process is repeated on all five stratified groups. Model's predictive ability is indicated by the average error of all five folds (https://www.mathworks.com/discovery/cross-validation.html).

Model evaluation

The bagging regression models omit on average 37% of the observations for each decision tree while generating bootstrap replicas of the dataset. These observations are called out-of-bag (OOB) observation and are used to evaluate the model performance. Our bootstrapped regression models for estimating monthly understory air temperature at daily average (T_{daily}), day-time (T_{dt}), and night-time (T_{nt}) durations performed well with coefficients of determination (R^2) ranging from 0.93 to 0.96, root mean square error (RMSE) from 0.48 to 0.65 °C and mean absolute errors from 0.75 to 1.0 °C. The predictive performance of ML regression model for T_{daily} is better compared to other models with T_{dt} model being the least accurate. In addition, spatial leave-one-out cross-validation analysis also gives more conservative estimates of the model used in this study (Fig. S10). The spatial leave-one-out cross-validation also demonstrated good prediction efficiency with accuracy parameters stabilizing after 40 km. More conservative estimates of each model performance and its variation along the buffer size can be seen in Fig. S10.

Independent validation of modelling results

We received additional data from four TOMST TMS logger installed in the Congo basin. These temperature observations were not used in training or testing of the model. We are using this new ground data for an independent assessment of the modelling results (i.e., T_{daily}, T_{dt}, and T_{nt}). Overall, we observed an $R^2 \geq 0.53$, RMSE≤0.72 °C, and MAE≤0.79 °C values for all three datasets. The scatter plot comparison of actual and modelled temperature is shown in Fig. S11. Important to note is that the values of RMSE and MAE are well within the range

depicted in Fig. S10. However, for these validation points, we observed an overall overestimation of 0.78±0.03 °C.

Supplementary Figures

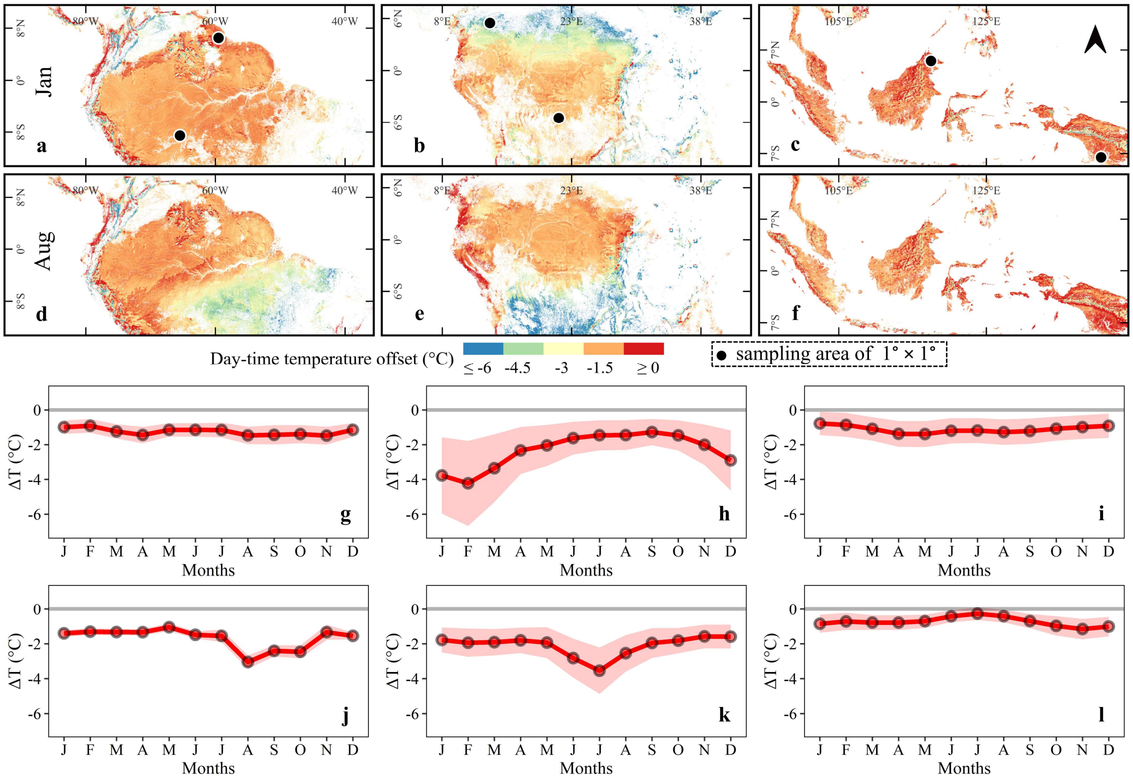

Fig. S1: Spatial and temporal variation of day-time temperature offsets. The offset (ΔT_{dt}) was calculated by subtracting open-air temperature (i.e., ERA5-Land) from modelled understory air temperature. Panels **a-f** show the pixel-level variations of ΔT_{dt} for two months of the year. To present monthly variation, six locations (each of the size 1°x1° area) were randomly selected on both sides of the equator in South America (**a**), Africa (**b**), and Southeast Asia (**c**). Panels **g-l** depict the intra-annual fluctuations of ΔT_{dt} at the selected locations. Panels **g-i** represent monthly ΔT_{dt} variation for the selected locations in the North while panels **j-l** represent ΔT_{dt} variation in the South. In line graphs, the shaded region around the solid (red) line represents the spatial variation within the selected block of each location.

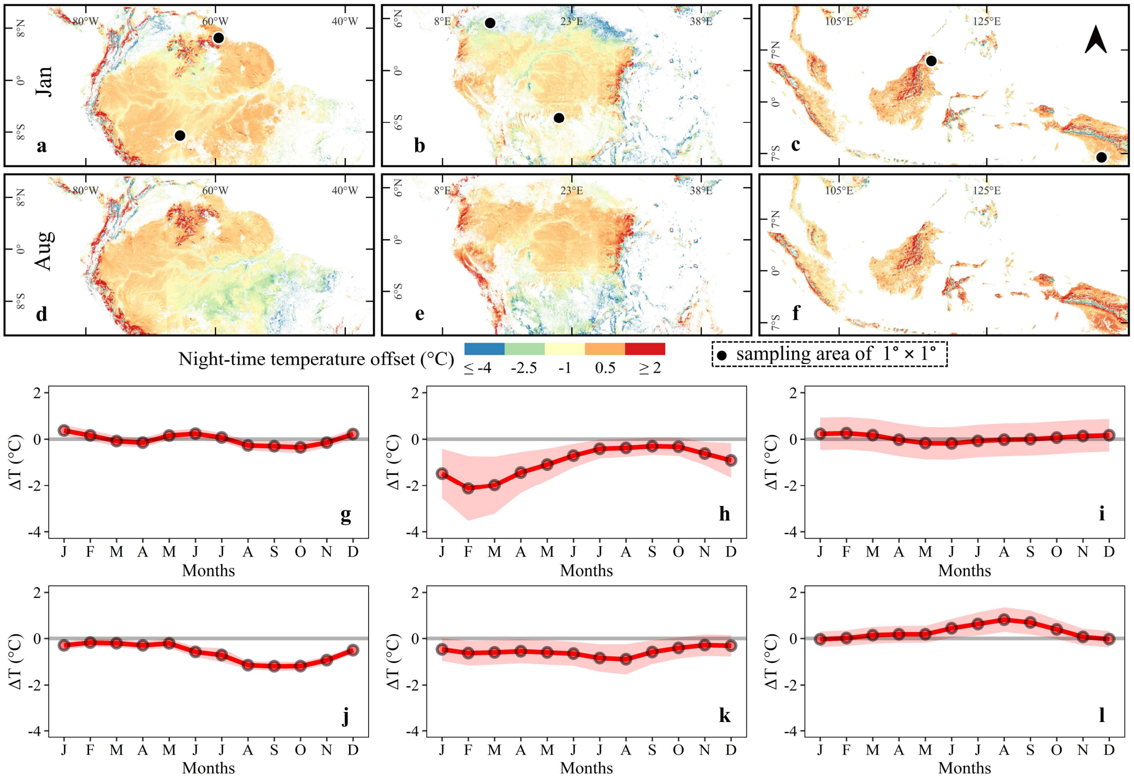

Fig. S2: Spatial and temporal variation of night-time temperature offset. The offset (ΔT_{nt}) was calculated by subtracting night-time open-air temperature (i.e., ERA5-Land) from modelled understory air temperature. Panels **a-f** show the pixel-level variations of ΔT_{nt} for two months of the year. To present monthly variation, six locations (each of the size 1°x1° area) were randomly selected on both sides of the equator in South America (**a**), Africa (**b**), and Southeast Asia (**c**). Panels **g-l** depict the intra-annual fluctuations of ΔT_{nt} at the selected locations. Panels **g-i** represent monthly ΔT_{nt} variation for the selected locations in the North while panels **j-l** represent ΔT_{nt} variation in the South. In line graphs, the shaded region around the solid (red) line represents the spatial variation within the selected block of each location.

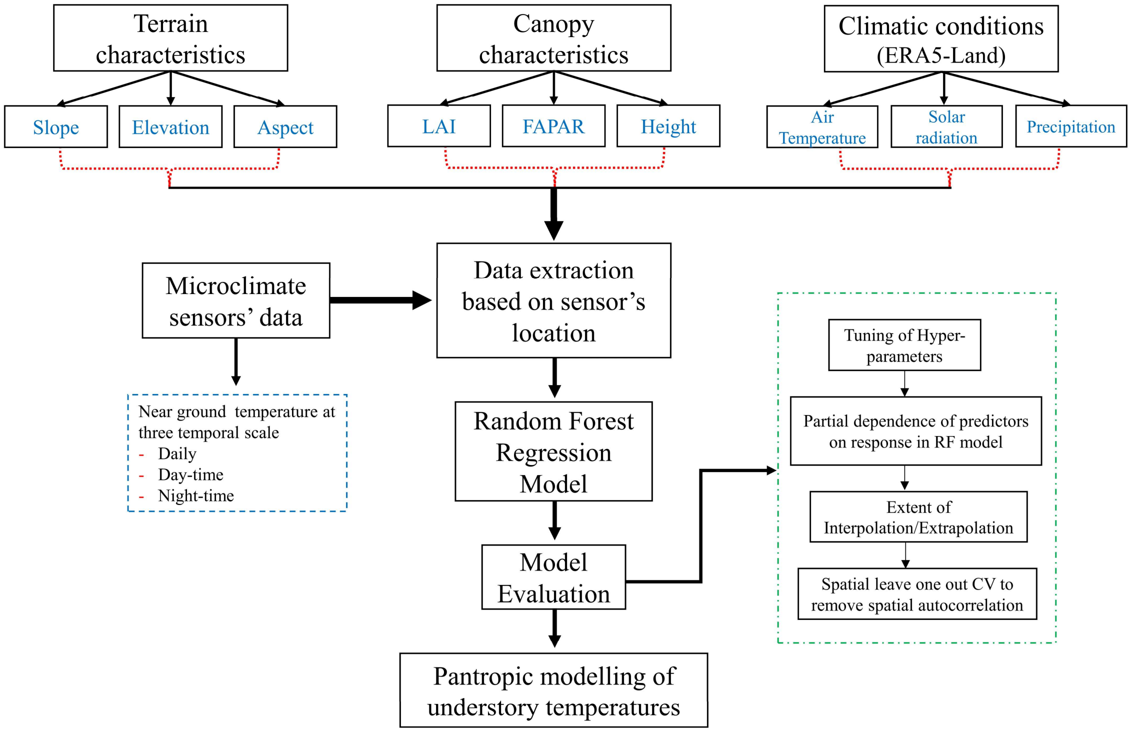

Fig. S3: Study method flow diagram.

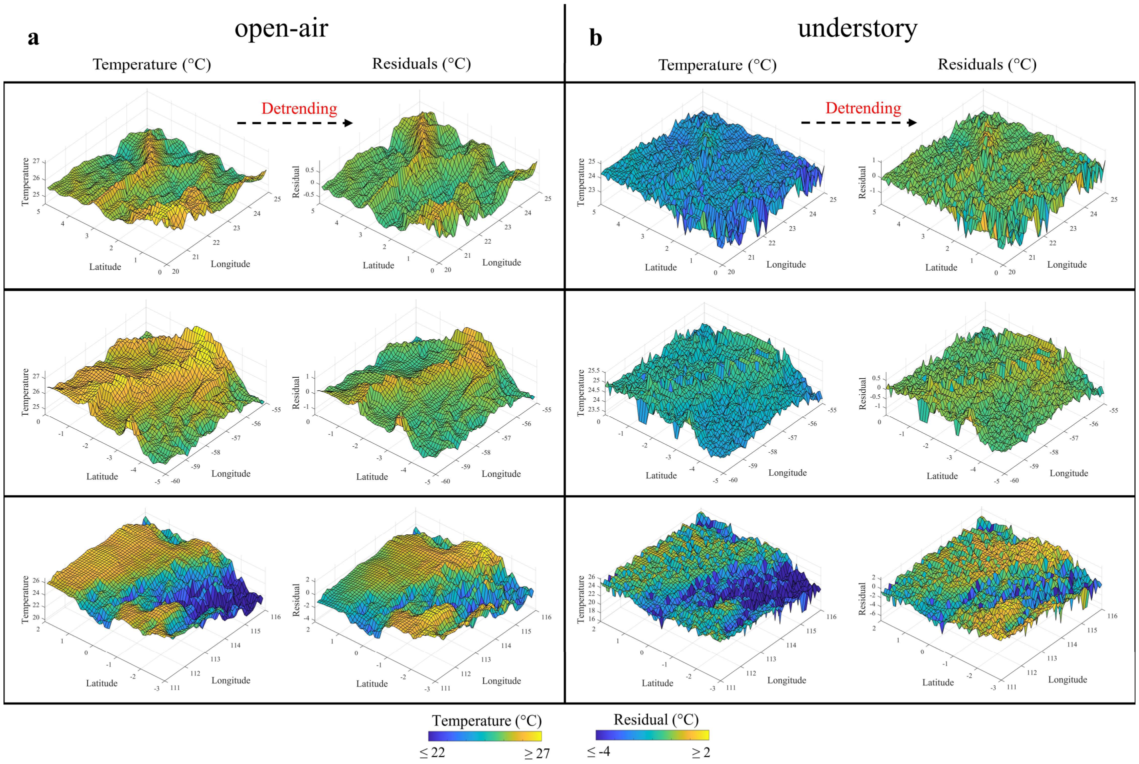

Fig. S4: Detrending process for semivariogram analysis. Panel **a** and **b** are for open-air and understory temperatures, respectively. The detrending was done by subtracting best fit surface from actual temperature data. Top row in each panel represents detrending of selected area from Africa, middle row represents area in South America, and last row represents area in Southeast Asia. The detrending of temperature surface generate residual data that was further used in semivariogram analysis.

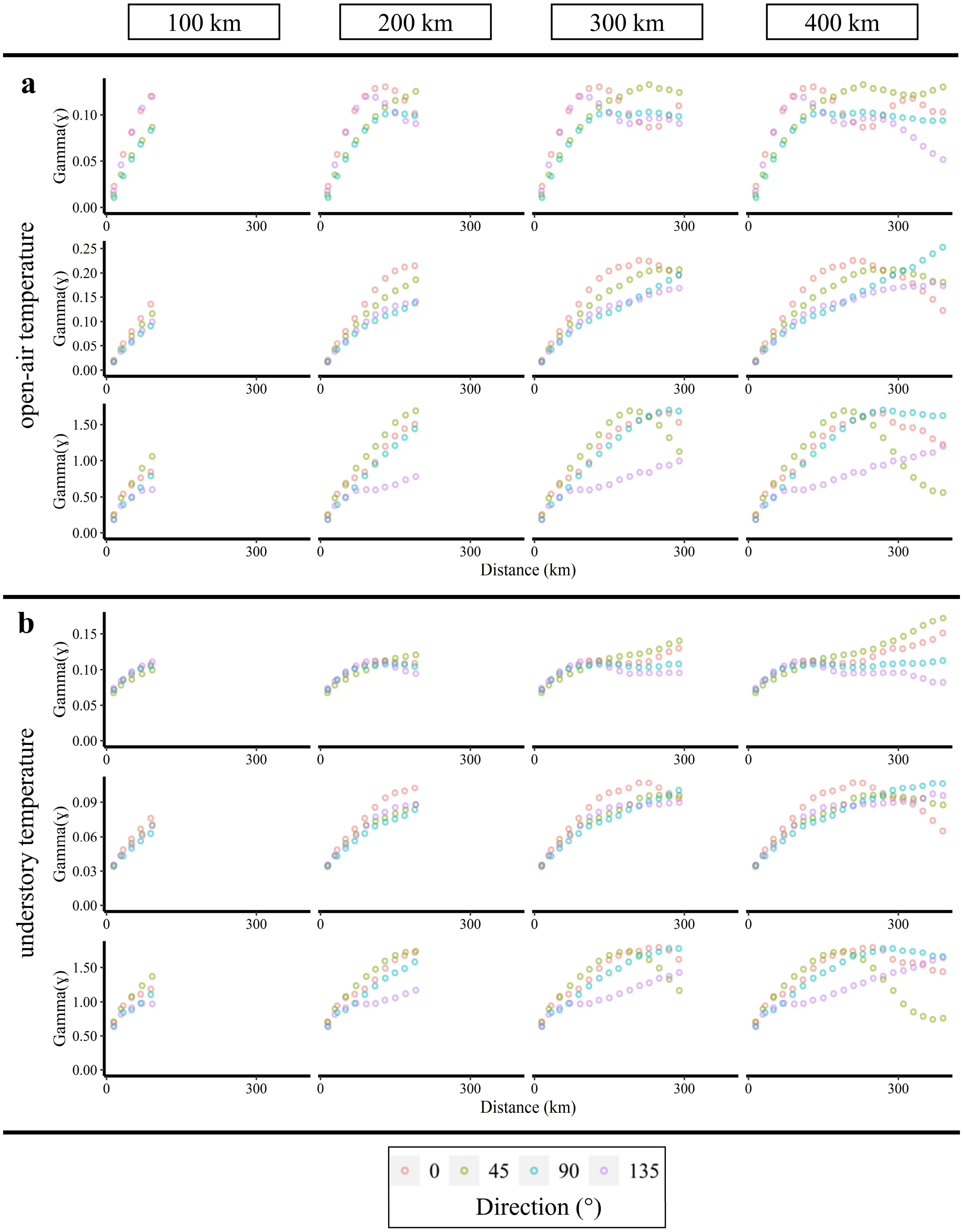

Fig. S5: Sample variogram behaviour under different combinations of distance and direction. Panel **a** and **b** show the semivariogram (ɣ) values for open-air and understory data under different combinations of distance and directions, respectively. The ɣ values for selected areas of Africa, South America and Southeast Asia are shown in top, middle and last rows of each panel, respectively. Horizontal direction (east-west) is depicted with 90° whereas 0° represents vertical (north-south) direction.

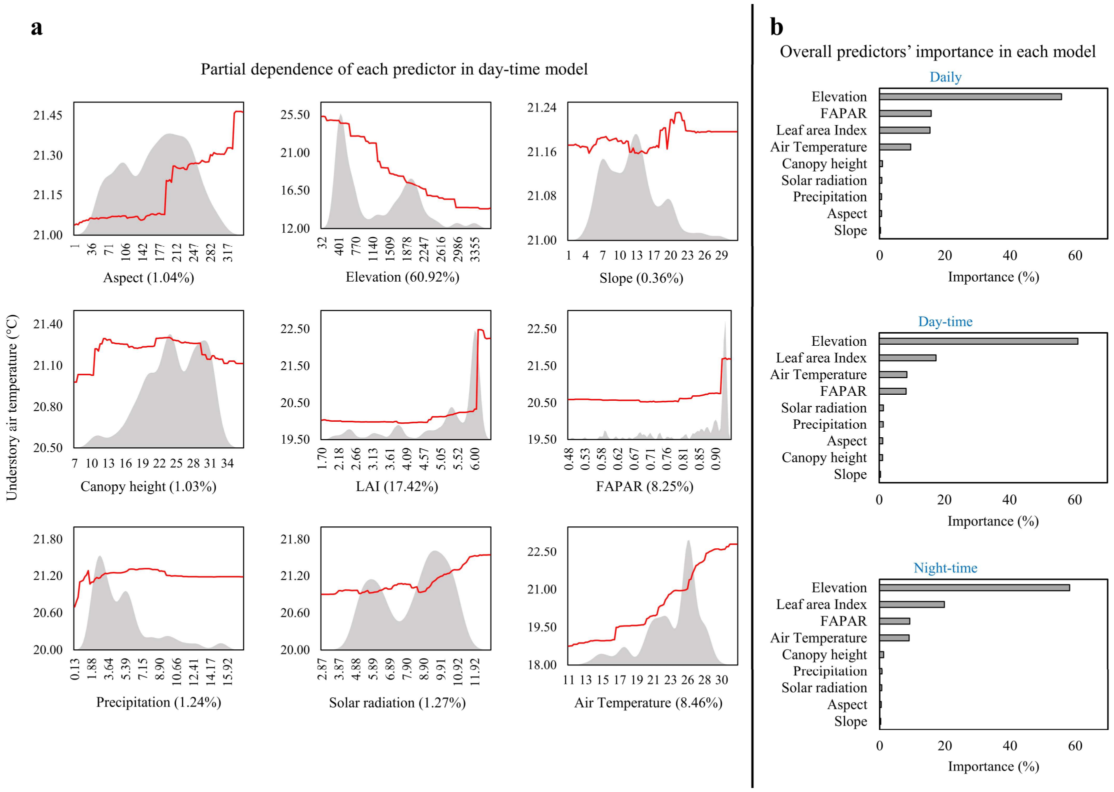

Fig. S6: Partial dependence plot. The plots in panel **a** indicate how much the response variable is affected by a certain predictor value, after accounting for the average effects of all other variables in the model. These partial dependence plots are for the model used for simulating day-time understory air temperature. The relative importance of each predictor in the model is reported between brackets. Gray backgrounds depict the distribution of the sample plots along that variable. Overall predictor's importance in each model is shown in panel **b**.

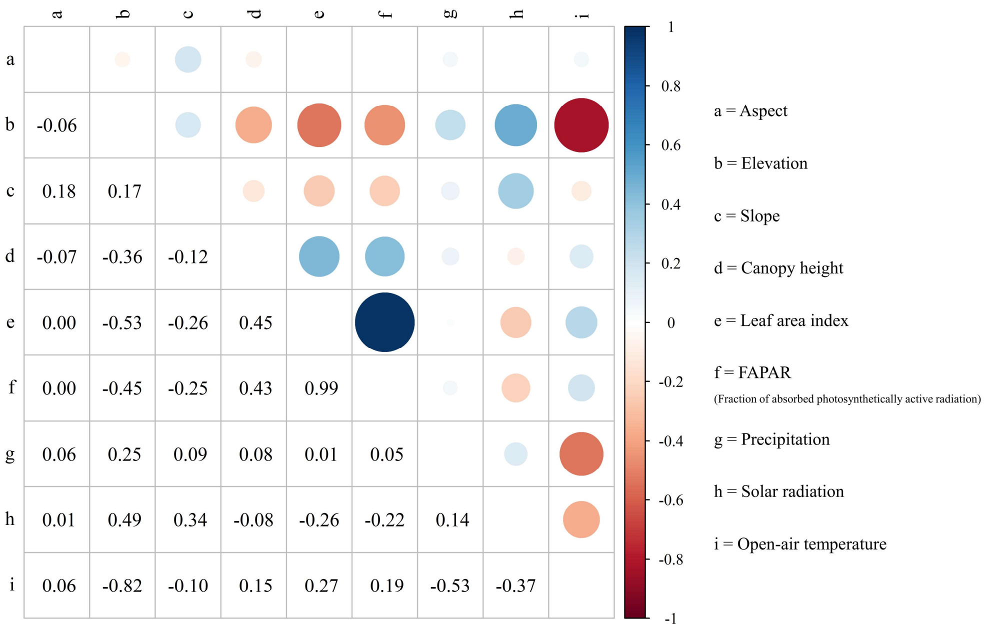

Fig. S7: Correlation matrix of explanatory variables. The correlation matrix containing all Pearson correlation coefficients (*r*) between all combinations of predictor variables. Note that the used machine learning model based on random forest is capable of handling multicollinearity between predictor variables.

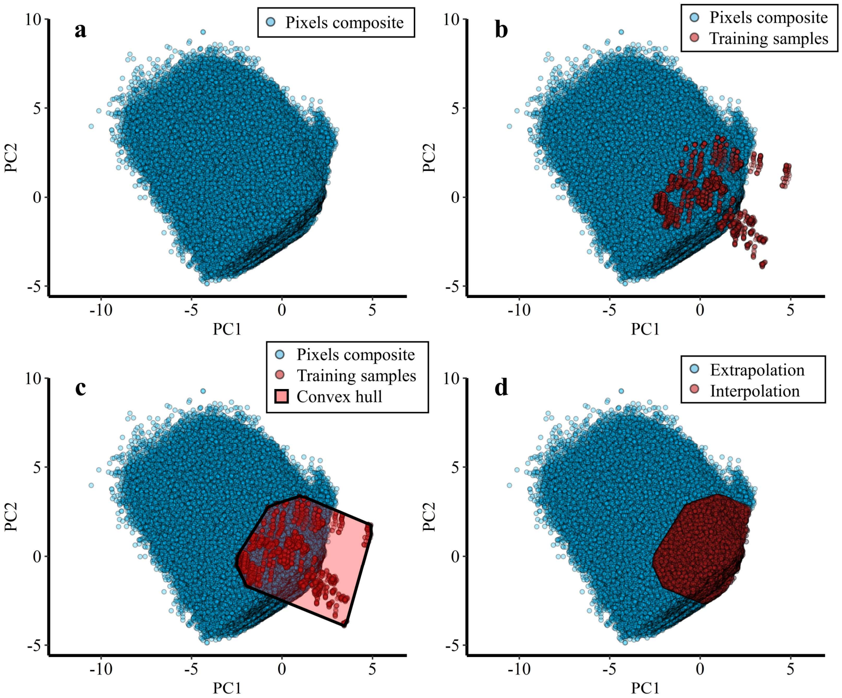

Fig. S8: Classification strategy for spatially explict model error. The visual representation of the adopted interpolation-extrapolation classification strategy of composite pixels in semi-multivariate space. After transforming predictors pixel values (**a**) and training data (**b**) into principle component (PC) space. Composite pixels falling within the convex hull encompassing the sampled data (**c**) are classified as interpolated, those outside as extrapolated (**d**). This process is repeated for all bivariate combinations of the selected PC axes. The final image is the proportion of covariates that are classified as interpolated. In our study dataset, first six PC axes explained >93% of variation represented by the dataset.

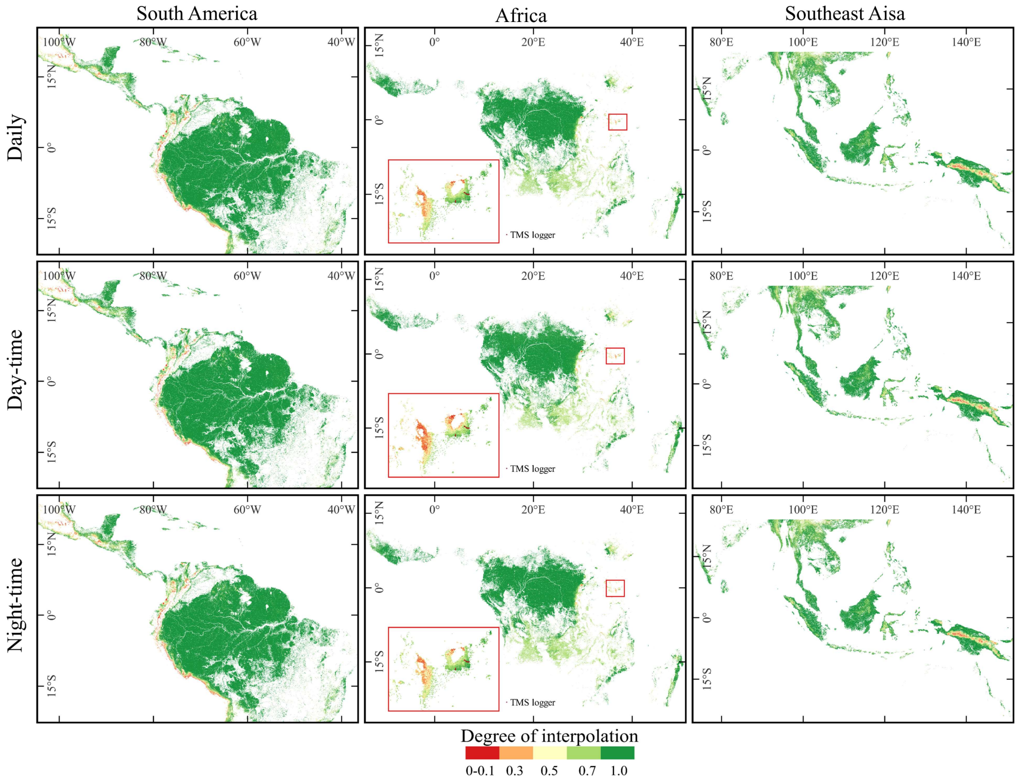

Fig. S9: Spatially explicit model errors. The pixel values closer to one indicate that values of predictor variables fell within the range of data covered by the in-situ measurement locations used to train the model. Low values indicate that the model extrapolated for many of the covariates at the specific location, as predictors values were not covered in the training dataset.

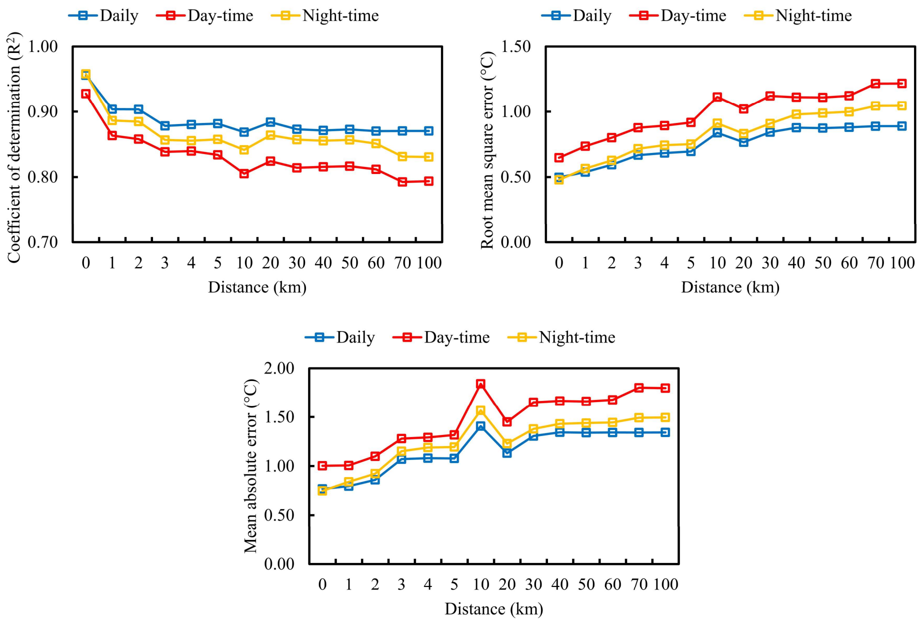

Fig. S10: Spatial leave-one-out cross-validation. This approach each time validates a model on data from one distinct location and trains a model on the remaining data. This is repeatedly done for each of our 180 locations. Because of potential spatial autocorrelation close to the validation location, this process is repeated with an increasing buffer around the validation location, each time excluding data points that fall within the defined buffer zone from the training data. This method allows assessing if the validation parameters stabilize, an indication of limited spatial autocorrelation.

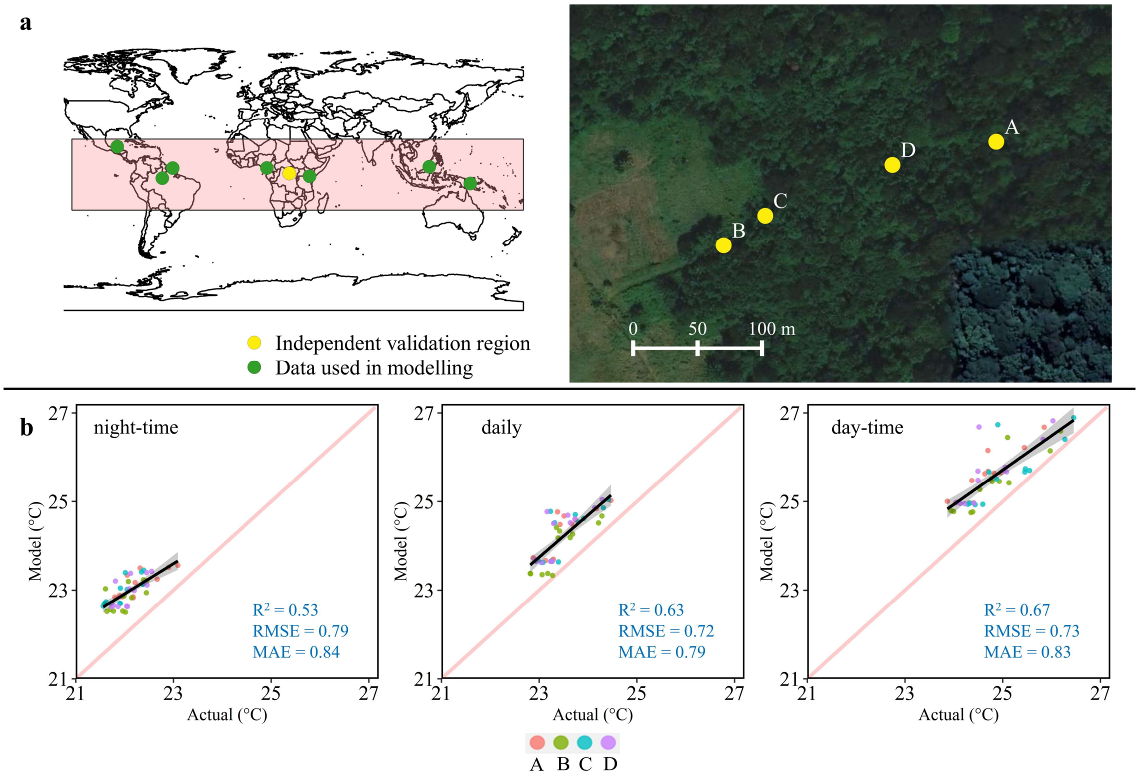

Fig. S11: Independent validation of modelling results. Panel **a** represents the locations of regions from where the microclimate sensors' data were used in modelling pantropical microclimate dataset (green points) and the region used for independent validation of the modelling results (yellow points). Panel **b** represents the monthly-level scatter plot comparision of understory measured and simulated temperature for four loggers A, B, C, and D, respectively.

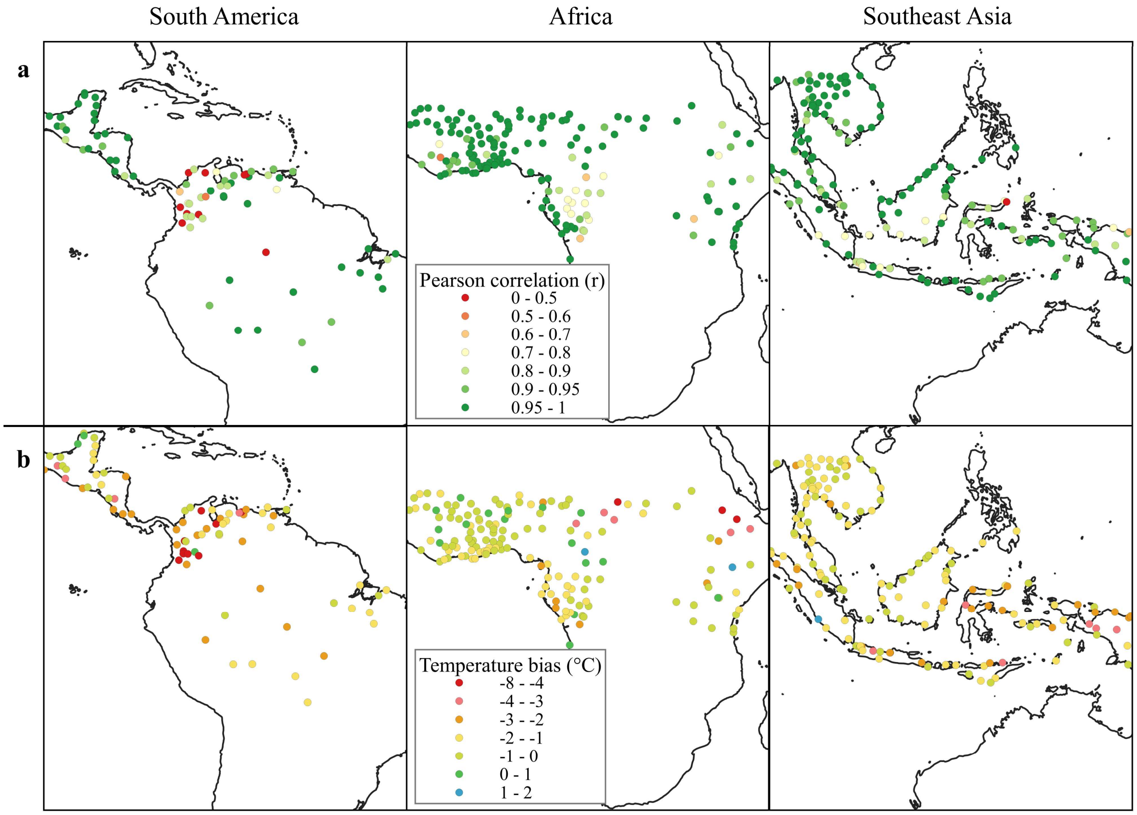

Fig. S12: Comparison of monthly temperatures from weather stations and their corresponding ERA5-Land pixel.

For this comparision, we selected active weather stations from the Global Historical Climatology Network – Daily (GHCN-Daily)[2] and employed the data between our study period (2000-2021). The weather station data were averaged to monthly scale and compared with corresponding ERA5-Land pixel temperatures. A total of 432 weather stations data from across tropics were used in this comparison. Fig. S12a shows the spatial distribution of statistically signification ($p<0.05$) value of Pearson correlation (r). Only 10 reanalysis pixels showed r<=0.5. Moreover, for each location, pixel-wise biases were calculated, and the spatial variability is shown in Fig. S12b. Overall, ERA5-Land temperature is underestimated by 1-2 °C. The mean bias was calculated using the formula: $bias = \frac{1}{n}\sum_{i=1}^{n}\left(T_{ERA_i} - T_{WS_i}\right)$ where n is the total observations of ERA5-Land (T_{ERA}) and weather station temperature (T_{WS}).

It's important to remember that weather station data has its own set of constraints. Even if we assume that weather station data is free from systematic and reporting errors (which likely is not the case for GHCN-Daily weather data as reported by Menne et al.,[79]), a weather station only records the weather conditions of a specific environment where it is installed. The stations used in our comparative analysis are mostly situated in urban areas. On the other hand, the ERA5-Land pixel depicts the average climate conditions across a 10 × 10 km area.

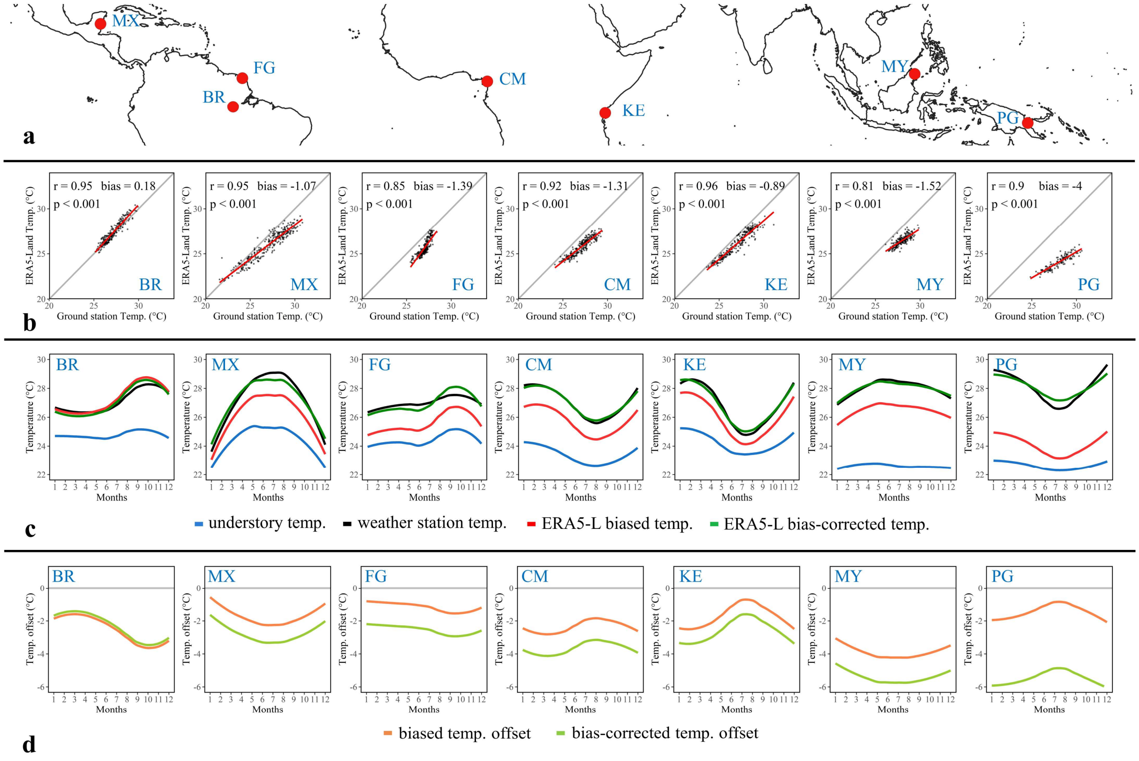

Fig. S13: Bias-correction in ERA5-Land temperature data and modelled temperature offsets.

We have employed a simple delta change approach[3,4] to bias-correct the ERA5-Land data and the temperature offset magnitudes. More detailed monthly-level correction using other approaches like multiple linear regression or quantile mapping approach can also be adopted.

To illustrate the example, we have used seven weather stations data from all those countries where TMS loggers were installed (Fig. S13a). Monthly average temperatures[5] were used to compare with corresponding ERA5-Land pixel and the mean bias (change factor) was calculated (Fig. S13b). The biased ERA5-Land temperature was adjusted using the calculated mean bias to estimate the bias-corrected reanalyzed temperatures (Fig. S13c). For example, the ERA5-Land pixel values in Mexico (MX) (Fig. S12b) were on average 1.07 (°C) lower when compared with weather station data. Therefore, 1.07 was added to the uncorrected ERA5-Land pixel values (as shown in red color line Fig. S13c) to get bias-corrected ERA5-Land temperature (i.e., green line, Fig. S13c). This corrected ERA5-Land temperature was then used to calculate actual temperature offsets as shown in Fig. S13d. The understory temperature shown in Fig. S13c is from a nearby forest area.

The estimated mean bias can also be directly added to the uncorrected temperature offsets (i.e., reported in this study) to derive bias-corrected temperature offsets. For example, estimated mean bias for MX is -1.07. This bias can be directly added to uncorrected temperature offsets (orange line, Fig. S13d) to get bias-corrected temperature offsets (light-green line, Fig. S13d).

Given our limited access to worldwide weather station data, especially the lack of access to average day-time and night-time temperature conditions, the end-users of the provided understory temperatures can perform local bias-correction using their national or regional weather station archives.

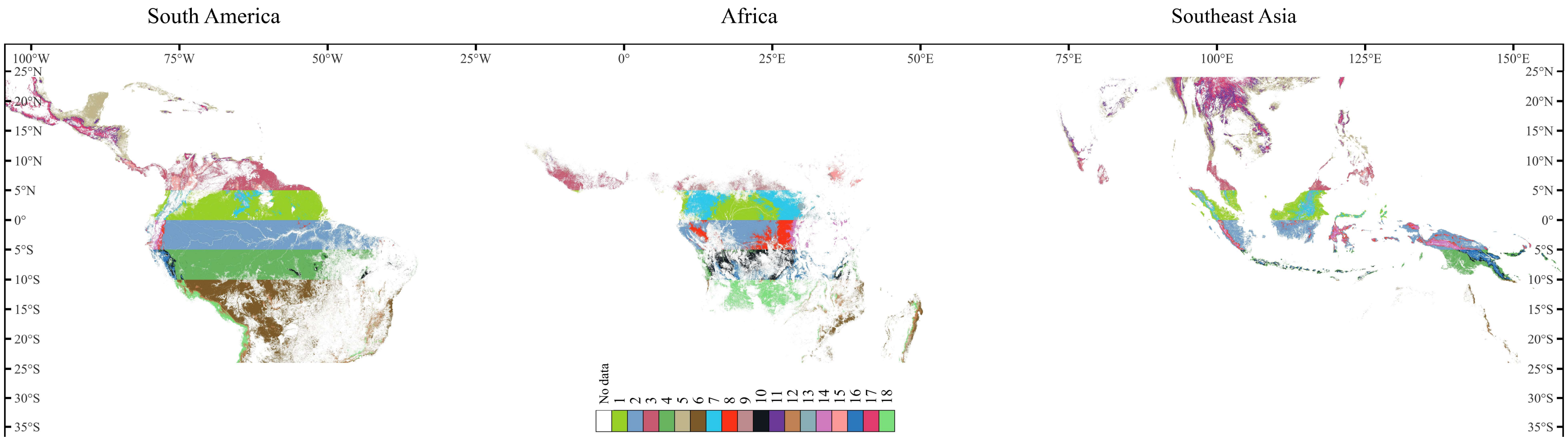

Fig. S14. Map showing the extent of different spatial zones use in this study to thoroughly examine modelling results. The description of each zone number can be found in Table S6.

Supplementary Tables

Table S1. The intra-annual variations of daily temperature offsets (ΔT_{daily}) (°C) for each spatial zone in three continents. For details of spatial zones, see Table S6 and Fig. S14. The positive offset values indicating warmer understory climate are in red and nagative offset values are in black.

Continents	Month	Spatial zones																	
		LLLN	LLLS	LLMN	LLMS	LLHN	LLHS	MLLN	MLLS	MLMN	MLMS	MLHN	MLHS	HLLN	HLLS	HLMN	HLMS	HLHN	HLHS
South America	Jan	-1.00	-1.04	-1.05	-0.86	-0.22	-1.34	-1.17	-0.89	-0.75	-0.86	0.03	-1.55	-2.78	-1.74	-2.99	-1.66	-2.23	-1.84
	Feb	-1.05	-1.01	-1.36	-0.84	-0.94	-1.24	-1.30	-0.87	-1.07	-0.83	-0.65	-1.46	-2.91	-1.79	-3.24	-1.62	-3.12	-1.67
	Mar	-0.96	-0.96	-1.42	-0.85	-1.37	-1.17	-1.22	-0.79	-1.20	-0.78	-1.48	-1.19	-2.76	-1.76	-3.26	-1.55	-4.23	-1.41
	Apr	-0.83	-1.03	-1.25	-0.95	-2.04	-0.98	-0.84	-0.67	-1.02	-0.80	-2.22	-0.57	-2.51	-1.69	-3.08	-1.53	-5.12	-0.72
	May	-0.96	-1.26	-0.96	-1.11	-2.09	-0.46	-0.44	-0.40	-0.72	-0.74	-2.19	0.52	-2.25	-1.41	-2.88	-1.29	-4.87	0.40
	Jun	-0.74	-1.12	-0.75	-1.31	-1.86	-0.37	-0.11	0.03	-0.48	-0.59	-1.86	1.08	-1.99	-0.87	-2.76	-0.91	-4.21	0.97
	Jul	-0.73	-1.21	-0.70	-1.60	-1.92	-0.52	-0.09	0.01	-0.43	-0.65	-1.80	1.11	-1.97	-0.74	-2.76	-0.84	-4.06	1.07
	Aug	-0.90	-1.49	-0.83	-1.97	-1.86	-1.38	-0.57	-0.43	-0.63	-1.20	-1.79	-0.02	-2.35	-1.19	-2.91	-1.35	-4.02	0.07
	Sep	-1.20	-1.64	-1.06	-1.82	-1.51	-1.66	-1.05	-0.86	-0.85	-1.49	-1.54	-1.00	-2.62	-1.65	-2.93	-1.71	-3.66	-0.94
	Oct	-1.39	-1.75	-1.13	-1.57	-1.13	-1.65	-1.26	-1.10	-0.86	-1.46	-1.03	-1.40	-2.58	-1.88	-2.77	-1.81	-3.13	-1.55
	Nov	-1.24	-1.58	-0.98	-1.20	-0.72	-1.31	-1.10	-1.09	-0.69	-1.19	-0.37	-1.27	-2.46	-1.92	-2.63	-1.83	-2.50	-1.69
	Dec	-0.97	-1.26	-0.91	-0.99	-0.30	-1.36	-1.00	-0.94	-0.57	-0.98	0.01	-1.44	-2.52	-1.77	-2.69	-1.70	-2.20	-1.81
Africa	Jan	-1.72	-1.14	-1.84	-1.63	-2.60	-1.85	-2.09	-1.25	-3.67	-1.68	-3.55	-1.55	-3.54	-2.82	-4.87	-3.00	-5.92	-3.10
	Feb	-1.91	-1.36	-2.21	-1.77	-3.53	-1.80	-2.29	-1.29	-4.59	-1.90	-4.62	-1.52	-4.03	-3.16	-5.61	-3.24	-6.99	-3.21
	Mar	-1.66	-1.25	-2.02	-1.62	-4.36	-1.59	-1.83	-1.27	-3.85	-1.86	-5.45	-1.33	-3.40	-3.06	-5.24	-3.16	-7.30	-3.11
	Apr	-1.37	-1.10	-1.74	-1.46	-4.58	-1.25	-1.55	-1.33	-2.79	-1.86	-5.31	-0.88	-2.85	-2.94	-4.35	-3.11	-7.12	-3.10
	May	-1.20	-1.09	-1.42	-1.42	-3.98	-0.38	-1.40	-1.34	-2.03	-2.45	-3.61	-0.36	-2.58	-3.01	-3.56	-3.97	-5.50	-3.71
	Jun	-1.02	-1.09	-1.10	-0.95	-2.71	0.48	-1.19	-1.34	-1.60	-2.68	-2.43	0.79	-2.33	-3.11	-3.19	-3.98	-4.16	-2.67
	Jul	-1.01	-1.08	-0.67	-0.47	-1.79	0.91	-0.95	-1.39	-1.26	-2.76	-1.64	1.47	-2.13	-3.16	-2.72	-3.90	-3.33	-2.36
	Aug	-0.95	-1.05	-0.58	-0.62	-1.53	0.47	-0.90	-1.24	-1.16	-3.04	-1.39	0.83	-1.99	-3.03	-2.90	-4.81	-3.26	-4.14
	Sep	-0.95	-1.04	-0.81	-1.09	-1.82	-0.71	-0.88	-1.09	-1.27	-2.60	-1.77	-0.29	-2.02	-2.90	-3.00	-4.75	-3.20	-5.57
	Oct	-0.99	-1.05	-1.10	-1.49	-2.15	-1.40	-0.88	-1.01	-1.40	-2.04	-2.15	-1.49	-2.10	-2.73	-2.94	-3.97	-3.56	-5.37
	Nov	-1.02	-1.01	-1.34	-1.53	-2.61	-2.08	-1.09	-0.92	-1.92	-1.66	-2.79	-1.88	-2.17	-2.51	-3.30	-3.27	-4.33	-3.82
	Dec	-1.21	-1.00	-1.53	-1.55	-2.53	-2.09	-1.55	-0.99	-2.92	-1.55	-3.23	-1.69	-2.69	-2.56	-4.02	-2.95	-5.25	-3.21
South Asia	Jan	-0.44	-0.56	-0.54	-0.70	2.12	-1.16	-0.23	-0.42	-0.81	-0.57	2.05	-1.43	-1.76	-2.03	-2.72	-2.31	0.84	-2.78
	Feb	-0.50	-0.58	-0.77	-0.63	1.24	-1.19	-0.34	-0.39	-1.02	-0.53	0.87	-1.50	-1.85	-1.97	-2.97	-2.26	-0.36	-2.82
	Mar	-0.62	-0.61	-1.09	-0.50	-0.29	-1.03	-0.42	-0.39	-1.35	-0.45	-0.80	-1.28	-1.94	-1.96	-3.33	-2.19	-2.34	-2.64
	Apr	-0.73	-0.60	-1.32	-0.46	-1.53	-0.57	-0.51	-0.38	-1.62	-0.44	-1.95	-0.63	-2.02	-2.02	-3.63	-2.22	-3.40	-2.29
	May	-0.85	-0.67	-1.34	-0.41	-1.85	0.10	-0.67	-0.47	-1.64	-0.42	-2.13	0.25	-2.20	-2.16	-3.59	-2.32	-3.59	-1.73
	Jun	-0.81	-0.58	-1.23	-0.22	-1.68	0.71	-0.62	-0.32	-1.51	-0.21	-1.93	1.33	-2.14	-1.99	-3.40	-2.18	-3.60	-1.06
	Jul	-0.69	-0.45	-1.10	-0.14	-1.58	1.41	-0.47	-0.11	-1.38	0.09	-1.75	2.21	-1.97	-1.74	-3.25	-1.85	-3.61	-0.39
	Aug	-0.68	-0.50	-1.06	-0.11	-1.46	1.62	-0.40	-0.05	-1.33	0.19	-1.64	2.42	-1.88	-1.64	-3.16	-1.71	-3.56	-0.18
	Sep	-0.67	-0.63	-0.96	-0.16	-1.26	1.05	-0.36	-0.16	-1.24	0.03	-1.51	1.62	-1.82	-1.71	-3.09	-1.75	-3.34	-0.65
	Oct	-0.59	-0.68	-0.78	-0.39	-0.90	0.22	-0.30	-0.34	-1.07	-0.25	-1.03	0.48	-1.75	-1.87	-2.94	-1.87	-2.63	-1.36
	Nov	-0.46	-0.59	-0.63	-0.59	-0.04	-0.37	-0.21	-0.38	-0.94	-0.41	-0.11	-0.34	-1.68	-1.94	-2.82	-2.03	-1.40	-2.01
	Dec	-0.45	-0.56	-0.57	-0.69	1.42	-0.97	-0.23	-0.41	-0.87	-0.53	1.48	-1.09	-1.75	-2.03	-2.77	-2.25	0.32	-2.55

Table S2. The intra-annual variations of day-time temperature offset (ΔT_{dt}) (°C) for each spatial zone in three continents. The positive offset values indicating warmer understory climate are in red and nagative offset values are in black.

Continents	Month	Spatial zones																	
		LLLN	LLLS	LLMN	LLMS	LLHN	LLHS	MLLN	MLLS	MLMN	MLMS	MLHN	MLHS	HLLN	HLLS	HLMN	HLMS	HLHN	HLHS
South America	Jan	-1.40	-1.51	-1.38	-1.42	-0.61	-2.02	-2.06	-1.75	-1.62	-1.60	-0.83	-2.31	-3.87	-2.58	-4.22	-2.65	-3.93	-2.73
	Feb	-1.41	-1.46	-1.64	-1.40	-1.38	-1.83	-2.22	-1.70	-1.99	-1.52	-1.62	-2.19	-4.02	-2.61	-4.48	-2.56	-5.00	-2.50
	Mar	-1.51	-1.42	-1.83	-1.41	-1.98	-1.79	-2.21	-1.56	-2.20	-1.49	-2.54	-1.99	-3.81	-2.61	-4.42	-2.51	-6.15	-2.33
	Apr	-1.37	-1.35	-1.66	-1.43	-2.57	-1.60	-1.73	-1.46	-1.95	-1.59	-3.32	-1.39	-3.40	-2.62	-4.01	-2.67	-7.01	-1.90
	May	-1.24	-1.36	-1.34	-1.37	-2.55	-0.98	-1.10	-1.19	-1.49	-1.52	-3.16	-0.30	-3.07	-2.44	-3.68	-2.56	-6.29	-0.88
	Jun	-1.12	-1.28	-1.12	-1.46	-2.09	-0.97	-0.80	-0.74	-1.21	-1.42	-2.57	0.18	-2.87	-1.91	-3.68	-2.27	-5.05	-0.41
	Jul	-1.10	-1.29	-1.10	-1.99	-2.16	-1.18	-0.83	-0.73	-1.20	-1.58	-2.46	0.23	-2.89	-1.82	-3.75	-2.23	-4.90	-0.31
	Aug	-1.46	-1.77	-1.27	-2.66	-2.21	-2.15	-1.48	-1.49	-1.52	-2.42	-2.52	-0.89	-3.47	-2.54	-4.00	-2.92	-4.86	-1.48
	Sep	-1.96	-2.10	-1.54	-2.34	-1.83	-2.53	-2.15	-2.09	-1.84	-2.69	-2.23	-2.03	-3.89	-3.05	-4.05	-3.27	-4.39	-2.44
	Oct	-2.24	-2.25	-1.58	-2.02	-1.54	-2.44	-2.38	-2.31	-1.82	-2.50	-1.70	-2.38	-3.70	-3.09	-3.74	-3.12	-4.21	-2.80
	Nov	-1.98	-1.93	-1.33	-1.59	-1.07	-2.03	-2.15	-2.21	-1.55	-2.09	-1.11	-2.04	-3.41	-3.00	-3.49	-3.03	-3.98	-2.68
	Dec	-1.49	-1.68	-1.21	-1.57	-0.75	-2.05	-1.90	-1.92	-1.36	-1.76	-0.87	-2.19	-3.46	-2.68	-3.73	-2.76	-3.94	-2.67
Africa	Jan	-2.63	-1.79	-2.74	-2.22	-4.09	-2.12	-3.17	-2.06	-5.09	-2.23	-4.87	-1.91	-4.96	-3.91	-7.04	-3.32	-7.72	-3.31
	Feb	-2.72	-2.00	-3.02	-2.36	-5.25	-2.09	-3.34	-2.28	-5.87	-2.55	-6.15	-1.90	-5.42	-4.37	-7.73	-3.77	-8.84	-3.49
	Mar	-2.42	-1.89	-2.68	-2.20	-6.16	-1.88	-2.75	-2.19	-4.70	-2.48	-6.99	-1.60	-4.57	-4.15	-6.96	-3.61	-8.91	-3.40
	Apr	-2.02	-1.77	-2.35	-2.02	-6.07	-1.47	-2.39	-2.07	-3.43	-2.47	-6.38	-1.05	-3.75	-3.89	-5.63	-3.67	-8.46	-3.78
	May	-1.85	-1.71	-2.05	-1.97	-4.79	-0.91	-2.18	-2.11	-2.66	-3.27	-4.16	-0.53	-3.46	-4.08	-4.60	-5.21	-6.33	-5.20
	Jun	-1.78	-1.68	-1.68	-1.94	-3.03	0.08	-1.83	-2.16	-2.15	-3.94	-2.87	0.51	-3.13	-4.33	-4.13	-5.51	-4.83	-4.12
	Jul	-1.66	-1.77	-1.17	-1.70	-1.95	0.45	-1.61	-2.34	-1.76	-4.17	-1.72	1.02	-2.95	-4.50	-3.57	-5.53	-3.71	-3.96
	Aug	-1.54	-1.64	-1.02	-1.64	-1.71	-0.48	-1.58	-2.01	-1.68	-4.19	-1.29	0.08	-2.82	-4.27	-3.85	-6.52	-3.68	-6.18
	Sep	-1.56	-1.66	-1.42	-1.95	-2.06	-1.41	-1.60	-1.86	-1.85	-3.48	-2.07	-1.15	-2.96	-4.09	-4.24	-6.35	-4.10	-8.03
	Oct	-1.58	-1.65	-1.70	-2.14	-2.46	-2.25	-1.56	-1.73	-2.06	-2.74	-2.69	-2.09	-2.93	-3.79	-4.28	-5.12	-4.59	-7.19
	Nov	-1.72	-1.60	-1.83	-2.13	-3.05	-2.83	-1.87	-1.63	-2.69	-2.28	-3.25	-2.42	-3.10	-3.48	-4.97	-3.86	-5.66	-4.62
	Dec	-1.85	-1.68	-2.30	-2.12	-3.79	-2.65	-2.42	-1.71	-4.16	-2.09	-4.33	-2.12	-3.97	-3.55	-6.10	-3.25	-6.97	-3.48
South Asia	Jan	-0.84	-0.98	-0.89	-0.92	1.46	-1.22	-0.93	-0.99	-1.20	-1.05	1.08	-1.86	-2.33	-2.54	-3.22	-2.94	-0.40	-3.35
	Feb	-0.99	-0.99	-1.24	-0.86	0.04	-1.25	-1.12	-0.97	-1.57	-0.98	-0.55	-1.85	-2.40	-2.44	-3.64	-2.81	-2.19	-3.31
	Mar	-1.08	-0.98	-1.69	-0.79	-1.51	-1.07	-1.23	-0.95	-2.02	-0.88	-2.31	-1.48	-2.54	-2.40	-4.19	-2.70	-4.25	-2.99
	Apr	-1.16	-0.99	-1.84	-0.78	-2.52	-0.56	-1.32	-0.94	-2.26	-0.88	-3.22	-0.69	-2.63	-2.45	-4.46	-2.76	-4.94	-2.58
	May	-1.28	-1.09	-1.65	-0.69	-2.35	0.14	-1.47	-1.05	-2.13	-0.82	-2.81	0.19	-2.80	-2.60	-4.19	-2.80	-4.41	-2.00
	Jun	-1.21	-0.94	-1.48	-0.44	-1.93	0.91	-1.34	-0.79	-1.91	-0.53	-2.25	1.15	-2.67	-2.33	-3.82	-2.59	-3.95	-1.32
	Jul	-1.13	-0.83	-1.41	-0.29	-1.77	1.50	-1.23	-0.61	-1.81	-0.25	-1.90	1.82	-2.53	-2.12	-3.72	-2.28	-3.72	-0.85
	Aug	-1.13	-0.95	-1.39	-0.28	-1.67	1.48	-1.24	-0.66	-1.80	-0.29	-1.83	1.68	-2.49	-2.08	-3.68	-2.24	-3.68	-0.84
	Sep	-1.09	-1.12	-1.29	-0.47	-1.52	0.84	-1.21	-0.87	-1.72	-0.57	-1.76	0.88	-2.42	-2.25	-3.63	-2.38	-3.50	-1.33
	Oct	-1.04	-1.18	-1.09	-0.79	-1.12	-0.03	-1.14	-1.10	-1.50	-0.90	-1.22	-0.18	-2.36	-2.50	-3.41	-2.62	-2.70	-2.18
	Nov	-0.90	-1.01	-0.96	-0.95	-0.22	-0.72	-0.97	-1.05	-1.31	-1.01	-0.31	-1.02	-2.26	-2.52	-3.24	-2.76	-1.68	-2.76
	Dec	-0.86	-0.99	-0.88	-0.96	1.30	-1.16	-0.96	-1.01	-1.23	-1.04	1.16	-1.69	-2.34	-2.57	-3.23	-2.94	-0.26	-3.25

Table S3. The intra-annual variations of night-time temperature offset (ΔT_{nt}) (°C) for each spatial zone in three continents. The positive offset values indicating warmer understory climate are in red and nagative offset values are in black.

Continents	Month	Spatial zones																	
		LLLN	LLLS	LLMN	LLMS	LLHN	LLHS	MLLN	MLLS	MLMN	MLMS	MLHN	MLHS	HLLN	HLLS	HLMN	HLMS	HLHN	HLHS
South America	Jan	-0.33	-0.44	-0.25	-0.19	0.69	-0.76	-0.07	0.13	0.31	0.00	1.33	-0.67	-1.86	-0.97	-1.86	-0.79	-0.78	-1.02
	Feb	-0.33	-0.36	-0.55	-0.13	0.08	-0.62	-0.19	0.15	0.02	0.04	0.76	-0.58	-1.96	-1.00	-2.06	-0.78	-1.37	-0.90
	Mar	-0.22	-0.28	-0.61	-0.14	-0.38	-0.52	-0.07	0.21	-0.07	0.11	-0.08	-0.25	-1.85	-0.96	-2.12	-0.70	-2.31	-0.55
	Apr	0.06	-0.26	-0.47	-0.19	-0.98	-0.10	0.21	0.33	0.01	0.16	-0.84	0.60	-1.70	-0.85	-2.11	-0.56	-3.16	0.32
	May	-0.08	-0.48	-0.19	-0.29	-1.15	0.40	0.48	0.60	0.18	0.27	-1.04	1.76	-1.50	-0.52	-2.01	-0.22	-3.28	1.38
	Jun	0.05	-0.47	0.01	-0.55	-1.15	0.51	0.81	1.00	0.40	0.45	-1.02	2.43	-1.22	-0.04	-1.84	0.20	-3.13	1.99
	Jul	0.03	-0.56	0.02	-0.83	-1.20	0.28	0.84	0.99	0.49	0.39	-0.97	2.46	-1.17	0.10	-1.78	0.28	-3.02	2.06
	Aug	-0.10	-0.75	-0.15	-1.13	-1.08	-0.45	0.52	0.79	0.39	0.05	-0.91	1.45	-1.34	-0.08	-1.82	-0.02	-2.96	1.33
	Sep	-0.44	-0.94	-0.29	-1.05	-0.78	-0.78	0.20	0.49	0.29	-0.25	-0.66	0.33	-1.51	-0.43	-1.81	-0.38	-2.68	0.37
	Oct	-0.59	-0.97	-0.32	-0.83	-0.44	-0.88	0.01	0.20	0.28	-0.33	-0.04	-0.20	-1.60	-0.79	-1.78	-0.66	-1.95	-0.44
	Nov	-0.45	-0.87	-0.21	-0.59	0.26	-0.78	0.04	0.11	0.35	-0.22	0.83	-0.32	-1.63	-0.91	-1.73	-0.76	-1.10	-0.79
	Dec	-0.33	-0.62	-0.12	-0.34	0.73	-0.81	0.05	0.12	0.42	-0.09	1.35	-0.58	-1.70	-0.92	-1.73	-0.76	-0.69	-1.01
Africa	Jan	-0.72	-0.51	-1.01	-0.92	-0.89	-1.23	-1.05	-0.34	-2.24	-0.94	-1.75	-1.09	-1.98	-1.68	-2.76	-2.14	-3.85	-2.51
	Feb	-1.06	-0.66	-1.35	-1.11	-1.42	-1.20	-1.39	-0.57	-3.25	-1.14	-2.67	-1.05	-2.46	-1.98	-3.54	-2.27	-4.82	-2.55
	Mar	-0.94	-0.64	-1.30	-1.03	-1.88	-1.03	-1.13	-0.55	-2.95	-1.11	-3.41	-0.90	-2.18	-1.97	-3.50	-2.23	-5.27	-2.41
	Apr	-0.77	-0.57	-1.16	-0.87	-2.21	-0.45	-0.88	-0.54	-2.21	-1.05	-3.64	-0.38	-1.84	-1.94	-3.02	-2.13	-5.29	-2.05
	May	-0.65	-0.56	-0.89	-0.64	-2.23	0.44	-0.71	-0.54	-1.51	-1.24	-2.59	0.54	-1.57	-1.95	-2.44	-2.34	-4.23	-2.03
	Jun	-0.48	-0.53	-0.40	0.11	-1.83	1.45	-0.38	-0.49	-0.98	-1.21	-1.66	1.97	-1.27	-1.90	-2.01	-2.05	-3.08	-0.69
	Jul	-0.30	-0.44	-0.10	0.62	-1.21	2.14	-0.11	-0.47	-0.58	-1.18	-1.13	2.82	-1.01	-1.87	-1.74	-1.87	-2.45	-0.25
	Aug	-0.16	-0.30	-0.04	0.44	-1.06	1.75	-0.04	-0.36	-0.48	-1.66	-1.00	2.25	-0.87	-1.81	-1.78	-2.75	-2.26	-2.09
	Sep	-0.13	-0.25	-0.12	0.00	-1.16	0.86	-0.02	-0.20	-0.51	-1.45	-1.12	1.01	-0.85	-1.70	-1.72	-2.97	-2.17	-3.64
	Oct	-0.21	-0.29	-0.32	-0.50	-1.34	-0.12	-0.06	-0.12	-0.59	-1.10	-1.31	-0.24	-1.03	-1.64	-1.63	-2.64	-2.32	-3.67
	Nov	-0.31	-0.28	-0.52	-0.70	-1.44	-0.84	-0.21	-0.04	-0.97	-0.87	-1.65	-0.95	-1.06	-1.50	-1.68	-2.27	-2.73	-2.83
	Dec	-0.56	-0.37	-0.90	-0.78	-0.95	-1.20	-0.59	-0.10	-1.58	-0.83	-1.58	-1.12	-1.33	-1.49	-2.08	-2.11	-3.28	-2.52
South Asia	Jan	0.24	0.04	0.12	-0.07	3.19	-0.83	0.45	0.16	-0.30	-0.07	3.40	-1.00	-1.23	-1.58	-2.28	-1.78	1.92	-2.51
	Feb	0.19	0.02	0.03	-0.05	2.78	-0.95	0.42	0.16	-0.32	-0.07	2.76	-1.20	-1.26	-1.55	-2.33	-1.78	1.43	-2.63
	Mar	0.07	0.00	-0.21	0.04	1.35	-0.83	0.36	0.17	-0.53	-0.02	1.06	-1.07	-1.34	-1.54	-2.57	-1.74	-0.24	-2.53
	Apr	-0.05	-0.01	-0.50	0.12	-0.18	-0.41	0.26	0.16	-0.87	0.02	-0.51	-0.49	-1.43	-1.59	-2.94	-1.73	-1.82	-2.21
	May	-0.20	-0.06	-0.72	0.15	-1.11	0.37	0.08	0.09	-1.09	0.01	-1.37	0.56	-1.62	-1.70	-3.12	-1.79	-2.71	-1.71
	Jun	-0.23	-0.01	-0.71	0.31	-1.36	1.20	0.03	0.17	-1.07	0.17	-1.58	1.74	-1.64	-1.62	-3.06	-1.69	-3.08	-1.01
	Jul	-0.09	0.17	-0.55	0.48	-1.34	1.95	0.23	0.42	-0.89	0.52	-1.52	2.73	-1.42	-1.35	-2.84	-1.39	-3.26	-0.31
	Aug	-0.01	0.23	-0.45	0.66	-1.21	2.31	0.39	0.58	-0.76	0.75	-1.36	3.21	-1.26	-1.17	-2.68	-1.19	-3.18	0.18
	Sep	0.03	0.17	-0.38	0.70	-0.98	1.93	0.46	0.57	-0.68	0.72	-1.17	2.66	-1.19	-1.15	-2.61	-1.15	-2.95	-0.06
	Oct	0.09	0.09	-0.22	0.44	-0.54	1.07	0.50	0.42	-0.56	0.47	-0.67	1.51	-1.14	-1.25	-2.51	-1.22	-2.31	-0.77
	Nov	0.18	0.06	-0.08	0.18	0.45	0.31	0.52	0.28	-0.47	0.24	0.41	0.47	-1.14	-1.39	-2.44	-1.40	-1.01	-1.50
	Dec	0.23	0.07	0.03	0.00	1.98	-0.36	0.47	0.19	-0.41	0.02	2.17	-0.38	-1.22	-1.53	-2.38	-1.64	0.88	-2.11

Table S4. The intra-annual variations of temperature range R_T for each spatial zone in the three continents. R_T values larger than 3 °C are highlighted in red.

Continents	Month	Spatial zones																	
		LLLN	LLLS	LLMN	LLMS	LLHN	LLHS	MLLN	MLLS	MLMN	MLMS	MLHN	MLHS	HLLN	HLLS	HLMN	HLMS	HLHN	HLHS
South America	Jan	2.66	2.24	2.71	2.13	2.76	2.55	2.25	2.04	2.43	2.29	2.64	2.63	2.31	2.05	2.35	2.04	2.62	2.25
	Feb	2.70	2.14	3.04	2.07	3.16	2.43	2.30	2.01	2.59	2.26	2.95	2.62	2.37	2.06	2.45	2.04	2.73	2.19
	Mar	2.54	2.08	3.24	2.11	3.45	2.65	2.23	1.99	2.60	2.24	3.21	2.64	2.34	2.05	2.51	2.07	2.89	2.23
	Apr	2.07	2.05	2.95	2.31	3.93	2.94	1.97	2.00	2.28	2.35	3.50	2.75	2.22	2.07	2.42	2.09	3.15	2.16
	May	2.12	2.30	2.46	2.64	3.67	3.07	1.92	2.07	2.08	2.56	3.12	2.59	2.15	2.03	2.36	2.04	3.07	2.00
	Jun	2.15	2.51	2.21	3.37	3.06	3.44	1.87	1.99	2.09	2.78	2.64	2.53	2.08	1.95	2.33	1.99	2.79	1.99
	Jul	2.21	2.69	2.24	3.63	3.17	3.57	1.87	2.06	2.11	2.88	2.66	2.50	2.06	1.94	2.35	1.99	2.71	1.99
	Aug	2.47	3.03	2.54	4.13	3.20	4.10	2.03	2.30	2.17	3.06	2.69	2.94	2.21	2.08	2.45	2.15	2.75	2.15
	Sep	2.94	3.13	2.90	3.94	3.12	3.98	2.29	2.42	2.28	3.15	2.60	3.23	2.36	2.20	2.51	2.21	2.71	2.29
	Oct	3.09	3.04	2.99	3.38	3.00	3.49	2.41	2.39	2.27	2.88	2.62	3.12	2.35	2.22	2.43	2.22	2.67	2.38
	Nov	2.93	2.97	2.76	2.90	2.80	2.88	2.25	2.31	2.17	2.54	2.59	2.69	2.26	2.22	2.37	2.17	2.59	2.29
	Dec	2.70	2.53	2.49	2.32	2.79	2.60	2.12	2.18	2.23	2.39	2.63	2.60	2.24	2.11	2.33	2.06	2.66	2.24
Africa	Jan	3.47	3.01	3.55	2.86	4.09	2.87	3.45	2.63	3.92	3.09	4.95	3.01	3.15	2.81	2.70	3.13	3.14	3.45
	Feb	3.34	2.94	3.40	3.00	3.86	2.85	3.34	2.72	3.82	3.22	4.73	2.98	3.23	2.91	2.87	3.10	3.20	3.51
	Mar	2.94	2.86	3.24	2.84	3.59	2.80	2.91	2.60	3.71	3.15	4.47	3.01	2.94	2.86	3.04	3.09	3.27	3.52
	Apr	2.79	2.63	3.05	2.55	3.44	2.74	2.59	2.49	3.33	3.18	4.44	3.18	2.79	2.73	2.85	3.11	3.24	3.54
	May	2.73	2.82	2.65	3.07	3.33	2.82	2.49	2.69	2.93	3.87	3.95	3.25	2.79	2.71	2.51	3.18	3.14	3.35
	Jun	2.65	3.31	2.04	3.34	2.72	2.79	2.44	3.14	2.73	4.26	2.89	2.81	2.82	2.63	2.28	3.25	2.62	3.26
	Jul	2.61	3.43	1.90	3.51	2.14	2.75	2.43	3.31	2.54	4.29	2.66	2.70	2.79	2.61	2.22	3.33	2.44	3.29
	Aug	2.42	2.99	1.89	3.41	1.98	3.19	2.38	2.99	2.51	4.11	2.62	3.17	2.82	2.70	2.22	3.33	2.39	3.63
	Sep	2.42	2.75	1.88	3.37	2.31	3.85	2.38	2.64	2.70	3.83	2.74	3.74	2.87	2.77	2.43	3.26	2.61	3.46
	Oct	2.44	2.61	2.28	3.29	2.81	4.01	2.37	2.50	2.84	3.43	3.20	4.10	2.84	2.73	2.61	3.21	2.94	3.52
	Nov	2.58	2.55	2.84	2.93	3.39	3.69	2.52	2.45	3.54	3.09	4.26	3.97	2.84	2.64	2.76	3.16	3.17	3.61
	Dec	3.40	2.67	3.34	2.77	3.87	3.14	3.17	2.46	3.94	3.03	4.92	3.46	2.98	2.70	2.71	3.17	3.10	3.47
South Asia	Jan	2.03	2.10	2.21	2.20	2.77	2.41	1.98	2.01	2.35	2.01	2.74	2.49	2.37	2.22	2.46	2.12	2.65	2.23
	Feb	2.32	2.19	2.72	2.14	3.29	2.25	2.13	2.03	2.71	2.00	3.43	2.33	2.68	2.26	2.78	2.09	3.15	2.13
	Mar	2.47	2.22	2.94	1.97	3.59	2.05	2.18	2.04	2.93	1.98	4.01	2.22	2.73	2.25	2.97	2.05	3.58	2.07
	Apr	2.47	2.18	2.84	1.95	3.59	1.99	2.18	2.01	2.89	2.01	3.89	2.20	2.70	2.19	2.92	2.03	3.77	2.03
	May	2.45	2.19	2.64	1.88	3.06	1.78	2.18	2.00	2.65	2.05	3.25	1.91	2.71	2.18	2.74	2.05	3.31	1.96
	Jun	2.33	2.04	2.44	1.65	2.53	1.72	2.14	1.93	2.46	2.00	2.65	1.71	2.69	2.12	2.57	2.02	2.83	1.86
	Jul	2.35	2.06	2.42	1.58	2.28	1.79	2.14	1.95	2.47	1.98	2.35	1.68	2.70	2.12	2.57	2.00	2.51	1.72
	Aug	2.46	2.26	2.50	1.74	2.25	1.96	2.18	2.06	2.55	2.09	2.34	1.75	2.73	2.25	2.66	2.05	2.54	1.78
	Sep	2.48	2.37	2.47	1.99	2.22	2.29	2.18	2.16	2.52	2.26	2.40	2.25	2.71	2.32	2.68	2.14	2.58	2.12
	Oct	2.38	2.39	2.29	2.28	2.30	2.59	2.13	2.20	2.41	2.33	2.51	2.73	2.58	2.38	2.57	2.23	2.59	2.37
	Nov	2.11	2.23	2.09	2.30	2.41	2.54	2.02	2.11	2.30	2.18	2.56	2.74	2.42	2.33	2.43	2.24	2.40	2.47
	Dec	2.00	2.09	2.04	2.22	2.46	2.54	1.96	2.02	2.26	2.05	2.48	2.70	2.31	2.22	2.37	2.15	2.23	2.41

Table S5. Number of TMS data loggers installed in each country and the duration of their temperature records used in this study.

Continental Area	Countries	No. of TMS data loggers	No. of Months with Temp. records	Duration of Temp. records	Average distance between data loggers
Americas	Brazil	63	8	Apr-2019 to Oct-2019	10-30 m at four locations 1-10 km apart
	French Guiana	16	17	May-2016 to Oct-2017	100-150 m at two location 130 km apart
	Mexico	5	8	Jan-2021 to Aug-2021	100 m
Africa	Kenya	35	26	Feb-2020 to Apr-2022	500-1000 m at three locations 5-35 km apart
	Cameroon	15	26	Jan-2015 to Feb-2017	200-500 m at two locations 30 km apart
South Asia	Papua New Guinea	31	14	Feb-2021 to Apr-2022	500-1000 m at two locations 10 km apart
	Borneo	15	25	Feb-2018 to Mar-2020	200 m

Table S6. The definition of each spatial zone and its characteristics. It also depicts the forest area percentage within each zone of the respective continent. Spatial extent of each zone is shown in Fig. S14.

No.	Spatial zone	Description	Elevation range (m)	Lattitude range (°)	Direction	Forest area (%)		
						South America	Africa	South Asia
1	LLLN	Low-elevation at low latitudes in the north	0-500	0-5	N	14.83	14.26	12.47
2	LLLS	Low-elevation at low latitudes in the south	0-500	0-5	S	22.29	15.57	17.62
3	LLMN	Low-elevation at mid latitudes in the north	0-500	5-10	N	6.00	5.72	4.73
4	LLMS	Low-elevation at mid latitudes in the south	0-500	5-10	S	19.06	1.66	8.24
5	LLHN	Low-elevation at high latitudes in the north	0-500	>10	N	6.17	0.38	17.89
6	LLHS	Low-elevation at high latitudes in the south	0-500	>10	S	15.72	4.07	1.19
7	MLLN	Mid-elevation at low latitudes in the north	500-1000	0-5	N	1.34	15.30	3.32
8	MLLS	Mid-elevation at low latitudes in the south	500-1000	0-5	S	0.33	7.78	3.02
9	MLMN	Mid-elevation at mid latitudes in the north	500-1000	5-10	N	1.11	4.27	1.07
10	MLMS	Mid-elevation at mid latitudes in the south	500-1000	5-10	S	0.72	6.96	2.02
11	MLHN	Mid-elevation at high latitudes in the north	500-1000	>10	N	1.73	0.09	11.82
12	MLHS	Mid-elevation at high latitudes in the south	500-1000	>10	S	3.20	3.03	0.29
13	HLLN	High-elevation at low latitudes in the north	>1000	0-5	N	0.70	1.40	1.84
14	HLLS	High-elevation at low latitudes in the south	>1000	0-5	S	0.48	2.12	3.22
15	HLMN	High-elevation at mid latitudes in the north	>1000	5-10	N	1.12	1.52	0.48
16	HLMS	High-elevation at mid latitudes in the south	>1000	5-10	S	0.67	3.72	3.01
17	HLHN	High-elevation at high latitudes in the north	>1000	>10	N	2.41	0.03	7.71
18	HLHS	High-elevation at high latitudes in the south	>1000	>10	S	2.11	12.12	0.04

Supplementary References

1. Pebesma, E., Graeler, B. & Pebesma, M. Package “gstat”. The Comprehensive R Archive Network.(CRAN), 1–0. (2015).

2. Menne, M. J., Durre, I., Vose, R. S., Gleason, B. E. & Houston, T. G. An overview of the global historical climatology network-daily database. Journal of atmospheric and oceanic technology 29, 897-910 (2012).

3. Hay, L. E., Wilby, R. L. & Leavesley, G. H. A comparison of delta change and downscaled GCM scenarios for three mountainous basins in the United States 1. JAWRA Journal of the American Water Resources Association 36, 387-397 (2000).

4. Ho, C. K., Stephenson, D. B., Collins, M., Ferro, C. A. & Brown, S. J. Calibration strategies: a source of additional uncertainty in climate change projections. Bulletin of the American Meteorological Society 93, 21-26 (2012).

5. Menne, M. J., Williams, C. N., Gleason, B. E., Rennie, J. J. & Lawrimore, J. H. The global historical climatology network monthly temperature dataset, version 4. Journal of Climate 31, 9835-9854 (2018).
